# Galactomannans: A Suitable Biopolymer to Produce Advanced Food Packaging

**DOI:** 10.1155/ijfo/9577986

**Published:** 2025-10-17

**Authors:** Elder dos Santos Araujo, Jéssica de Matos Fonseca, Alcilene Rodrigues Monteiro, Germán Ayala Valencia

**Affiliations:** ^1^ Department of Chemical and Food Engineering, Federal University of Santa Catarina, Florianópolis, Santa Catarina, Brazil, ufsc.br

**Keywords:** biodegradable films, coatings, food packaging, galactomannan, gums

## Abstract

Galactomannans (GMs) have promising food packaging applications as edible films and coatings. These natural polysaccharides offer an environmentally friendly and sustainable alternative to conventional nonbiodegradable and nonedible plastic materials. By forming biopolymeric matrices with desirable mechanical, thermal, and barrier properties, GM not only enhances the preservation and quality of food products but also addresses growing concerns about plastic waste and environmental pollution. The versatility and compatibility of GM with other biopolymers and bioactive compounds further expand their applicability, positioning GM‐based films and coatings as a promising solution for advancing sustainable packaging technologies in the food industry. Given the increasing demand for sustainable food packaging, this review is aimed at consolidating and presenting the current research on the use of GMs in food packaging applications. Despite the potential of GM‐based materials, studies on this topic remain limited. Therefore, this review provides a comprehensive overview of existing research while highlighting knowledge gaps and unexplored opportunities. By addressing these limitations, this work offers a novel perspective on the application of GM in sustainable packaging, contributing to the advancement of innovative and environmentally friendly solutions.

## 1. Introduction

Global population growth, urbanization, and globalization have significantly increased the demand for food, amplifying challenges related to food safety, distribution, and availability. Packaging plays a vital role in safeguarding food and beverages from quality degradation throughout distribution, sale, and consumption [1].

Plastic is widely chosen for food packaging due to its inherent characteristics, including lightweight nature, strength, durability, accessibility, convenience, and cost‐effectiveness [2]. Moreover, its excellent mechanical strength, thermal stability, and corrosion resistance [1], along with its structural flexibility and tunable properties, further enhance its suitability for this application [3].

In recent decades, plastic films have been widely used to protect food from external threats such as heat, moisture, and microorganisms [4]. However, this widespread use comes with significant drawbacks. The escalation of plastic pollution remains a global concern, and although the “reuse, reduce, and recycle” approach provides some relief, it falls short in addressing the pervasive reliance on plastics [5]. As consumer awareness of environmental sustainability increases, so does the demand for more eco‐friendly packaging solutions [4].

Biodegradable plastics hold a limited market share due to their lower performance, higher cost, and reduced legislative focus compared to conventional materials (e.g., petroleum‐derived polymer, glass, and metal) [6]. However, these materials have strong potential to contribute to sustainable development, supporting a green economy, reducing greenhouse gas emissions, and promoting the valorization of residual biomass [5].

A notable trend in the food packaging industry is the shift to renewable resources, particularly bio‐based hydrocolloids. Edible films and coatings have gained significant attention as a promising technology to extend the shelf life of foods while complementing traditional packaging methods [7]. Biodegradable plastic materials not only support the green economy and reduce greenhouse gas emissions, but advances in modifying natural polymers have also improved their mechanical and physical properties, increasing their potential for industrial and packaging applications [5].

These bio‐based materials from renewable resources have emerged as viable alternatives, reducing reliance on nonrenewable petrochemical packaging and minimizing environmental impact. These innovations extend shelf life, preserve nutritional quality, and address concerns related to waste management and sustainability in the food industry [8].

In this context, galactomannans (GMs) have been used to manufacture edible films and coatings [9, 10], and as gel formers [11], thickeners [12], viscosity modifiers [13], stabilizers, and fat substitutes in several food formulations [14, 15], as well as to manufacture water absorbents [16]. One of the key characteristics of GM is their ability to easily form hydrogen bonds and produce materials with appropriate film‐forming properties [17].

GM is a complex polysaccharide characterized by a *β*‐(1 → 4)‐linked d‐mannan backbone with single d‐galactose side chains attached via *α*‐(1 → 6) linkages [7, 17]. The mannose‐to‐galactose (M/G) ratio and branching degree vary depending on the GM source. For instance, the GM extracted from *Gleditsia triacanthos* exhibits a polymerization degree of 224 and a branching degree of 0.24, whereas that from *Adenanthera pavonina* has a higher polymerization degree of 475 and a branching degree of 0.60 [18]. This polysaccharide is photostable, nontoxic, biocompatible, and it does not have nutritional value, being desired for food, pharmaceutical, and packaging industries when compared with other biopolymers such as starch and gelatin, which are broadly used for similar packaging applications [19].

It is estimated that approximately 90–100 thousand tons of GM are consumed annually. Most of this consumption is attributed to guar gum (GG), with approximately 70–80 thousand tons, followed by locust bean gum (LBG), accounting for 12–14 thousand tons. However, global GM consumption figures can vary significantly depending on the data source [17].

Edible packaging based on polysaccharides has potential to be used in foods and replace nonbiodegradable packaging [7]. Since the food packaging industry heavily relies on plastics, recycling alone is insufficient to ensure environmental sustainability [5]. To date, no review has systematically analyzed the use of GM for packaging applications. Therefore, this review is aimed at exploring the literature about the packaging application of GM in the food industry. The use of GM is aligned with the United Nations’ 2015 Sustainable Development Goals by promoting eco‐friendly alternatives to conventional plastics [20].

## 2. Research About GMs and Packaging Applications

**Figure 1:**
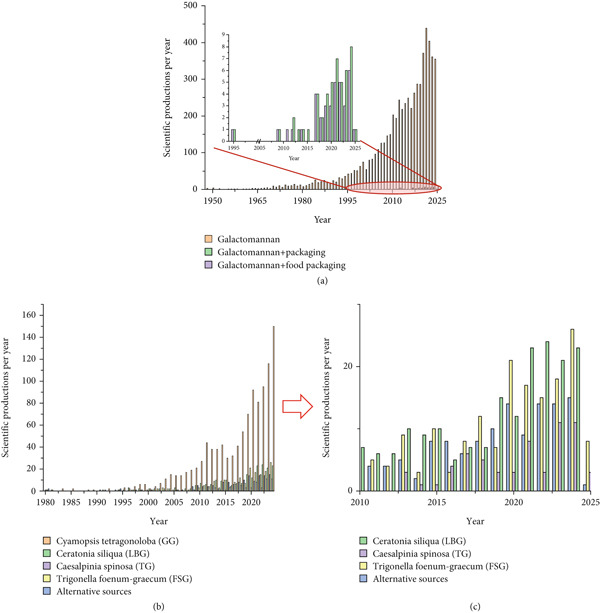
(a) Bibliometric data search containing galactomannan at the Scopus database platform (http://www.scopus.com). (b, c) Bibliometric data research about the main galactomannans used for packaging applications and using the same database platform.

A bibliographic search in the Scopus database was conducted using the keywords “galactomannan,” “galactomannan + packaging,” and “galactomannan + food packaging” (Figure 1a). Although numerous studies on GM exist in the literature, the first publication addressing their application in food packaging appeared only in 1995. Significant scientific production on this topic began around 2010, marking the start of a notable and consistent increase in research efforts, reflecting the growing interest in sustainable and functional packaging solutions.

## 3. GM Sources

**Figure 2:**
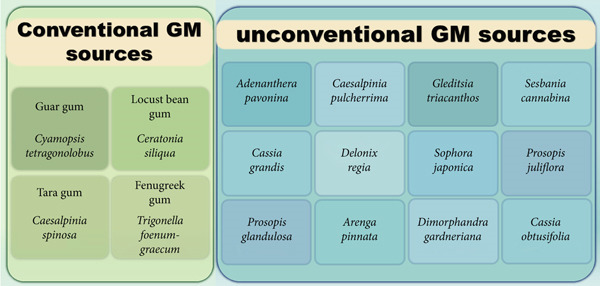
Conventional and unconventional galactomannan (GM) sources.

Four major sources of seed GMs are LBG (*Ceratonia siliqua*), GG (*Cyamopsis tetragonoloba*), tara gum (TG) (*Caesalpinia spinosa*), and fenugreek (*Trigonella foenum-graecum*) (Figure 1b,c). Although only GG and LBG are considered important from an industrial point of view [17], GM can be extracted from numerous alternative species from different botanical families (Figure 2), such as *Cassia absus*, *Cassia emarginata*, *Cassia fistula, Cassia leptocarpa, Caesalpinia cacalaco*, and *Delonix regia* in Caesalpiniaceae*; Desmanthus illinoensis* and *Leucaena glauca* in Mimosaceae; and *Sophora japonica, Genista scoparia*, and *Lotus corniculatus* in Fabaceae [17]. These species are unconventional GM sources with potential industrial applications.

Based on the literature analyzed in this study, 19.2% utilized GM extracted from *Adenanthera pavonina*, 13.5% from *Caesalpinia pulcherrima* and *Gleditsia triacanthos*, 9.6% from *Cyamopsis tetragonolobus* (GG) and *Sesbania cannabina*, 7.7% from *Ceratonia siliqua* (LBG) and *Trigonella foenum-graecum* (fenugreek), 5.8% from *Cassia grandis*, and 1.9% from *Delonix regia*, *Sophora japonica*, *Prosopis juliflora*, *Prosopis glandulosa*, *Arenga pinnata*, *Dimorphandra gardneriana*, and *Cassia obtusifolia*.

### 3.1. GG

GG or cluster bean is a polygalactomannan extracted from the endosperm of the leguminous plant *Cyamopsis tetragonolobus*; it is the most consumed GM in the world, representing between 70% and 88.9% of global GM consumption (around 70–80 thousand tons) [17]. Its structure features a backbone of *β*‐d‐mannopyranosyl units linked by (1–4) bonds, with single *α*‐d‐galactopyranosyl units attached to roughly every second unit of the main chain through (1–6) linkages. It exhibits exceptionally high viscosity even at very low concentrations (≤ 1% w/v) in aqueous solutions [21].

It has an M/G ratio of 2, which leads to a distinct morphology due to steric hindrance, preventing the polymer chains from intertwining via interchain hydrogen bonding and therefore limiting the formation of a strong network. When the M/G ratio increases from 2 to 4, as in LBG, the basic chemical structure of GM remains unchanged. However, this higher ratio allows for tighter packing of the polymer chains, reduces crystallinity, and creates a more compact and uniform film morphology, resulting in improved mechanical properties [22].

The guar plant is an annual summer legume primarily cultivated in India and eastern Pakistan, with smaller scale production in regions such as South and Central America, Africa, Brazil, Australia, and the Southwestern United States. It grows to about 1 m in 5 months and produces pods slightly smaller than the carob. Each guar pod contains up to 10 seeds, with the endosperm comprising about 35% of the total weight [17].

The economic significance of guar cultivation increased with the discovery of GM, making India the leading producer, supplying around 80% of the global demand with an annual yield of 1 to 1.25 million tons. While also cultivated in Australia, China, Pakistan, and Africa, GG has become a valuable trade commodity, with industrial applications representing approximately 45% of its total demand [23].

The commercial production process of GG can also be used for LBG and TG, beginning with the separation of the endosperm from the seed coat and germ. The endosperm is then ground and sifted into a fine flour. Further purification can be applied by repeated washings with alcohol; most commercial gums contain more than 80% GM, and the quality and purity of the final gum product depend largely on how completely the endosperm is separated and hulled during processing [17, 24].

Due to its unique properties, GG is widely used in the food industry as a gelling agent, thickener, stabilizer, emulsifier, binding agent, flocculent, fracturing agent, foam stabilizer, and antifouling agent. In its partially hydrolyzed form, it also serves as a soluble dietary fiber, with proven prebiotic effects that help reduce blood glucose and cholesterol levels. In addition, GG is applied in the production of low glycemic index foods due to its dietary fiber content. Recent innovations include its use in the creation of biodegradable films for food packaging and as a wall material for flavor encapsulation [21, 25].

GG‐based packaging offers beneficial properties for preserving the quality of packaged products. Films made from GG and pullulan, enriched with loquat leaf extract, effectively maintain the freshness and nutritional value of Chinese water chestnuts, demonstrating performance comparable to or even superior to nonbiodegradable plastic (PE) [26].

Active and intelligent packaging made from GG can be created by combining it with polyvinyl alcohol (PVA), silver nanoparticles, and pokeweed betacyanins. This combination provides films with excellent rigidity, thermal stability, and enhanced antioxidant and antibacterial properties. Additionally, chitosan/dialdehyde GG hydrogels with extract of pomegranate peel demonstrate remarkable antioxidant and antimicrobial effects, making them ideal for use as antibacterial pads in packaging applications [27].

It has also been investigated as a tackifier in biodegradable adhesives. When mixed with other natural components such as bael gum, these adhesives exhibit improved mechanical peel strength and biodegradability, making them ideal for multilayer films and reinforced paper bags [28].

A shortage of LBG in the 1940s prompted the development of GG as an alternative. This innovation is aimed at meeting the demand for thickening and stabilizing agents in various industries, particularly in food and pharmaceuticals. Among all GM, LBG and GG are the only ones of significant industrial importance [17].

### 3.2. LBG

LBG is a GM extracted from the endosperm of seeds of the carob tree (*Ceratonia siliqua*). It is the second most consumed GM in the world, corresponding to 12%–15.6% (between 12 and 14 thousand tons per year) [17].

The carob pod contains approximately 10% seed by weight. The endosperm, often called “splits,” consists of two spherical halves that enclose the germ. These splits are ground into flour, which produces a cloudy solution when dissolved in water. To obtain a clearer product, the flour is dispersed in hot water, and the insoluble particles are removed by filtration using diatomaceous earth. The clarified solution is then precipitated with isopropyl alcohol, washed with alcohol, pressed, dried, ground, and sieved. The final product is a white to creamy powder that should produce a clear solution when dissolved in water [17].

It is commonly used as an additive across various industries, including food, pharmaceuticals, paper, textiles, oil drilling, and cosmetics. The industrial use of LBG is largely attributed to its capacity to form hydrogen bonds with water molecules, providing excellent thickening, stabilizing, and emulsifying properties. Additionally, LBG offers health benefits, such as aiding in the management of diabetes, regulating bowel movements, and reducing the risk of heart disease and colon cancer, due to its function as a dietary fiber [29, 30].

Preclinical studies have demonstrated that LBG exhibits very low toxicity, primarily due to its indigestible nature. It is commonly used as a thickener in infant formulas for the therapeutic management of uncomplicated gastroesophageal reflux (GER) [30]. It can also be used to control root‐knot incognita in tomato plants [31].

LBG has an M/G ratio of 4, consists of mannose (73%–86%) and galactose (14%–27%), and has a molecular weight ranging from 50,000 to 3,000,000 [30]. This provides improved film‐forming properties compared to GG. LBG films exhibit higher elastic modulus, tensile strength, and elongation at break. These findings indicate that the chemical structure of LBG promotes denser packing of the polymer chains, resulting in films that are stronger and more flexible. This makes LBG an attractive option for applications requiring improved mechanical properties [22]. While fenugreek and GG are readily dissolved in cold water, heating is necessary to reasonably solubilize LBG in water [17].

Studies have reported that LBGs are suitable for packaging applications and also present good compatibility and stability with other materials, such as *κ*‐carrageenan, sunflower seed proteins, and sodium alginate, increasing mechanical strength, barrier properties, and thermal stability and reducing swelling rates [32–34].

Active films made from sodium alginate, LBG, and lecithin, enriched with daphnetin, exhibited excellent mechanical, optical, and barrier properties. Additionally, they demonstrated antibacterial activity (*Shewanella putrefaciens* and *Pseudomonas fluorescens*), as well as notable antioxidant effects [34].

Additionally, smart packaging films based on LBG and PVA containing anthocyanins or betacyanins can be produced to indicate shrimp freshness. These films change color in response to pH or ammonia levels, providing a visual indication of spoilage, in addition to exhibiting antioxidant activity [35, 36].

LBG is classified as INS/E 410 according to the food additive numbering system. Internationally, the Joint FAO/WHO Expert Committee on Food Additives (JECFA) evaluated the safety of LBG in 1981. Based on its very low toxicity, observed mainly in in vitro and animal studies, the committee assigned an acceptable daily intake (ADI) of “not specified,” reflecting the minimal risk associated with its consumption [30].

### 3.3. TG

Although GG and LBG are the two most used GM in the food industry, a third member of this family, TG, has also been explored for its potential applications, offering properties that fall between those of GG and LBG. It is obtained from the endosperm of the tara seed, which comes from the leguminous plant *Caesalpinia spinosa*, native to Peru and neighboring regions [37]. TG is one of the most extensively studied polysaccharides in recent decades, with applications in nutraceutical, pharmaceutical, and biomedical fields, as well as in food packaging and controlled drug release systems [38].

TG is known for its high viscosity, excellent water‐holding capacity, and strong protective colloidal properties. TG also exhibits interfacial tension activity and demonstrates good resistance to acids and salts, making it suitable for various applications in the food industry [37]. In addition to being affordable, nontoxic, and biodegradable [38].

While TG is moderately soluble in cold water, complete dissolution usually requires heat, similar to LBG [37]. It has an M/G ratio of 3, providing advantageous film‐forming properties that surpass those of GG in terms of strength and flexibility. Although TG has a higher M/G ratio than GG, this does not alter its fundamental chemical structure; however, it facilitates a denser packing of polymer chains and reduces crystallinity, resulting in smoother, more compact films with fewer nodular structures [22].

Similar to LBG, TG produces films with improved elasticity and tensile strength, suggesting that its intermediate M/G ratio achieves an ideal balance between structure and functionality [22]. Due to these characteristics, TG is considered a promising material for secure food packaging, preserving food quality and protecting bioactive compounds [38].

Studies have shown that TG stands out as an excellent candidate for the production of edible films due to its excellent mechanical and barrier properties, making it ideal for food packaging applications [22, 39]. The incorporation of oleic acid into TG enhances the water resistance and hydrophobicity of these edible films while also improving their thermal stability, making them highly promising for packaging applications [40].

Furthermore, TG can be blended with other materials to enhance the performance and functionality of the resulting films. It demonstrates strong compatibility with materials like PVA, forming films with enhanced mechanical strength, hydrophobicity, and improved barrier properties against oxygen and UV radiation [39]. Incorporating curcumin into this matrix yields smart films capable of colorimetric responses to ammonia exposure, making them effective visual indicators for monitoring shrimp spoilage [41].

The incorporation of microcrystalline cellulose and grape skin extracts (*Vitis vinifera* var. Pinot Noire) into TG also allows the development of smart films capable of detecting pH changes, making them effective indicators for monitoring milk spoilage [42]. TG has been effectively used for microencapsulation of bioactive compounds [43, 44].

Despite these studies, its most common use is in food formulations, like a thickener [45] and stabilizer [46], and can form thermoreversible gels in the case of a mixture of *κ*‐carrageenan or xanthan [47]. TG has been effectively used as an additive to improve the rheological and textural characteristics of gluten‐free bread formulations [48] and a modifier of the rheological and textural properties of tapioca starch [49]. TG also can substitute conventional stabilizers in ice cream without compromising technological and sensory quality [50], and its application in low‐calorie and reduced‐fat foods has been growing globally [38].

The TG market is also growing rapidly, as it offers a cost‐effective alternative to GG. It can be safely used in the food industry for a variety of applications, as it meets the necessary criteria to be classified as a GRAS (generally recognized as safe) food ingredient [38].

### 3.4. Fenugreek Gum

Fenugreek (*Trigonella foenum-graecum*) is an annual plant that belongs to the Fabaceae family, cultivated as a semiarid crop in North Africa, the Mediterranean, India, and Canada. Fenugreek seeds contain approximately 26.8% GM, which shares similar properties to the soluble fiber found in guar seeds and *psyllium* husk. Fenugreek seed gum (FSG) has a balanced M/G ratio of 1:1. This equilibrium, combined with the uniform distribution of galactose and mannose fractions, enhances hydration, making FSG the most soluble among seed gums [51, 52].

Studies indicate that FSG can be used for the manufacture of environmentally friendly packaging systems. Furthermore, FSG has antioxidant and antifungal activity [52]. These properties can be further enhanced by incorporating antimicrobial agents such as penicillin, resulting in significant antimicrobial activity against common foodborne pathogens, including *Escherichia coli*, *Staphylococcus aureus*, and *Candida albicans*, along with notable antioxidant effects. This makes them particularly valuable for food packaging applications, helping to extend the shelf life of meat and cheese while ensuring product safety [53].

Also, FSG‐based nanocomposite can be produced. Films reinforced with nanoclays (montmorillonite, halloysite, and Nanomer) at concentrations up to 5% have shown great potential for antimicrobial food packaging applications. These films exhibit strong antimicrobial activity against foodborne pathogens, including *Listeria monocytogenes*, *E. coli* O157:H7, *S. aureus*, and *Bacillus cereus* [54].

In addition to nanocomposites, biocomposites with excellent packaging properties can be developed using pectin, FSG, microfibrillated cellulose (MFC), and plant extracts. The resulting composite films demonstrate significant antioxidant and antibacterial activity against *S. aureus* and *E. coli*, highlighting their strong potential for commercial food packaging applications, particularly in extending the shelf life of fresh‐cut carrots [55].

Therefore, FSG films demonstrate great potential as food packaging materials due to their flexibility, mechanical strength, biodegradability, and antioxidant and antimicrobial activities [52, 53]. However, the use of FSG, like TG, is limited mainly due to its availability and cost. Also, compared to LBG and GG, which are more widely produced and economically accessible, FSG and TG are less common, making them less favorable for industrial applications [17].

## 4. GMs and Packaging Applications

### 4.1. Biodegradable Films

**Figure 3:**
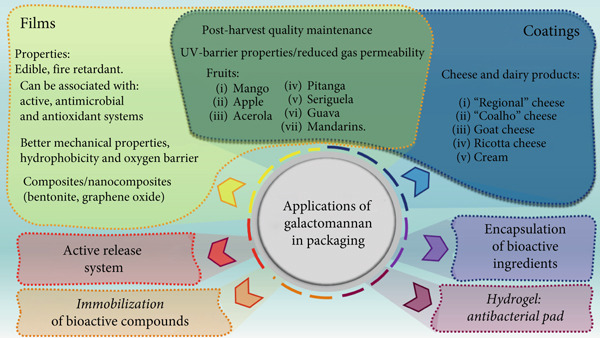
Main galactomannan packaging applications.

There is a growing interest in replacing current packaging films with bio‐based films without compromising mechanical properties and hydrophobicity [56]. GM is widely used in the production of edible films and coatings for food applications (Figure 3), playing a crucial role in improving microbial safety and preserving food quality. Its structural characteristics directly influence the film‐forming capacity [57].

These materials form protective barriers that help reduce contamination, extend shelf life, and maintain the sensory characteristics of products, contributing to sustainable and functional solutions in the food industry [7]. GMs with a reduced galactose content, such as LBG and modified GG, form films with enhanced tensile strength and greater elongation at break [57].

GM films are well‐suited for food packaging applications (Table 1), owing to their high tensile strength, uniform surface morphology, and excellent flexibility (Tables 2, 3, and 4). These properties ensure the films’ durability and adaptability, making them effective in protecting and preserving food products while offering a sustainable alternative to traditional packaging materials [58].

**Table 1:** Galactomannan packaging applications.

**Galactomannan (GM) source and formulation**	**Packaging type, main result, and application**	**Ref.**
Tara gum (*Caesalpinia spinosa*) and PVA	Films The authors produced composite films with adequate packaging properties, including UV barrier; however, these materials were not applied in food systems	[38]
Chitosan, GM (*Adenanthera pavonina* L.), sodium acetate, and glycerol	Films and coatings Composite films had antimicrobial activity against gram‐negative and gram‐positive bacteria. These films were not applied to food systems	[58]
GM (*Adenanthera pavonina*, *Cyamopsis tetragonolobus*, *Caesalpinia pulcherrima*, *Ceratonia siliqua*, and *Sophora japonica*) and glycerol	Films The authors concluded that mannose/galactose ratios influence galactomannan‐based edible films’ final properties. These films were not applied to food systems	[59]
GM (*Sesbania cannabina*), arabinoxylan (waste wheat straw), and bentonite nanosheets	Films The authors produced materials like papers with improved barrier properties. These films were not applied to food systems	[60]
Nacre‐like borated GM (*Sesbania cannabina* seeds) with graphene oxide	Films The authors produced materials with hydrophobic surfaces and improved mechanical and barrier properties. These films were not applied in food systems	[61]
Guar gum (*Cyamopsis tetragonolobus*) acrylated	Films Acrylated guar gum films had UV‐barrier properties. These films were not applied in food systems	[62]
Borated cross‐linked galactomannan (*Sesbania cannabina*), bentonite, and graphene oxide	Films The authors produced films with fire retardant property and low oxygen permeability. These films were not applied in food systems	[63]
GM (*Gleditsia triacanthos*), natural extract (*Gleditsia triacanthos*), and glycerol	Films The authors developed films with antioxidant properties. These films were not applied in food systems	[64]
GM (*Gleditsia triacanthos*), soy protein isolate, and glycerol	Films The authors produced composite films with adequate packaging properties; however, these materials were not applied in food systems	[65]
GG (*Cyamopsis tetragonoloba*), loquat leaf extract, pullulan, and glycerol	Films The films showed preservation of the quality of Chinese water chestnuts after 4 days of storage, better or at least comparable to nonbiodegradable plastic wrap (PE)	[25]
Sodium alginate, LBG (*Ceratonia siliqua*), lecithin, glycerol, and daphnetin	Films Active film with antioxidant and antibacterial properties, with potential for the development of food‐grade packaging material, however, these materials were not applied in food systems	[33]
*κ*‐Carrageenan and GM GG (*Cyamopsis tetragonoloba*) or LBG (*Ceratonia siliqua*)	Films Authors claim that *κ*‐carrageenan‐GM film has potential in packaging applications; however, these materials were not applied in food systems	[31]
LBG (*Ceratonia siliqua*), PVA, glycerol, and anthocyanins or betacyanins	Films Smart packaging suitable for indicating the freshness of shrimp	[34, 35]
TG (*Caesalpinia spinosa*), oleic acid, glycerol, and sorbitol	Edible packaging materials with good barrier against water vapor, however, these materials were not applied in food systems	[39]
TG (*Caesalpinia spinosa*), PVA, curcumin, and glycerol	Films Smart films for shrimp spoilage indicators	[40]
TG (*Caesalpinia spinosa*), PVA, and glycerol	Films Good blend film for use in film packaging industries, however, these materials were not applied in food systems	[38]
TG (*Caesalpinia spinosa*), microcrystalline cellulose, glycerol, and grape skins extracts (*Vitis vinifera* var. Pinot Noire)	Films Smart films capable of monitoring milk deterioration	[41]
FSG (*Trigonella foenum-graecum*), glycerol, and penicillin	Films Antimicrobial (against *E. coli*, *S. aureus*, and *Candida albicans*) and antioxidant active film with potential application for maintaining the properties of chicken meat and cheese	[52]
FSG (*Trigonella foenum-graecum*) montmorillonite, halloysite, Nanomer, and glycerol	Films Nanocomposite films with strong antimicrobial activity (against *Listeria monocytogenes*, *Escherichia coli* O157:H7, *Staphylococcus aureus*, and *Bacillus cereus*), however, these materials were not applied in food systems	[53]
Pectin, FSG, microfibrillated cellulose (MFC), plant extracts (*Vitis vinifera* Linn seed, *Bauhinia variegata* Linn leaf, and *Annona squamosa*), and glycerol	Films Biocomposites with antioxidant and antibacterial activity (against *S. aureus* and *E. coli*), applied to the extending the shelf life of fresh‐cut carrots	[54]
GG (*Cyamopsis tetragonoloba*), Laponite, and glycerol	Composite film with improved barrier properties that improve the shelf life of ready‐to‐eat strawberries	[66]
GM (*Caesalpinia pulcherrima* and *Adenanthera pavonina L.*) and glycerol	Coatings Coatings were applied on acerola (*Malpighia emarginata*), cajá (*Spondias lutea*), mango (*Mangifera indica*), pitanga (*Eugenia uniflora*), and seriguela (*Spondias purpurea*) to extend the fruit shelf life	[9]
Chitosan, GM (*Gleditsia triacanthos*), agar (*Gracilaria birdiae*), corn oil, glycerol, sorbitol, Tween 80, and lactic acid	Coatings These materials were applied in *Regional Saloio* cheese	[10]
GM (*Adenanthera pavonina* L.) and glycerol	Coatings The coating reduced weight loss and delaying softening and thus in maintaining quality of mangoes (*Mangifera indica* cv. Tommy Atkins)	[67]
Mesquite gum (*Prosopis juliflora* (Sw.), reflective additives (hydroxyapatite, montmorillonite, and bentonite clays)	Coatings The authors observed that coated mangoes (*Mangifera indica* L.) had less sunburn effect	[68]
GM (seeds of *Adenanthera pavonina* L. and *Caesalpinia pulcherrima*), collagen, and glycerol	Coatings The shelf life of mangoes (*Mangifera indica*) and apples (*Malus domestica*) was extended with the use of coating materials	[69]
GM (*Gleditsia triacanthos*), glycerol, corn oil, and nisin	Coatings Edible coatings reduced the microbial growth and weight loss of Ricotta cheese, extending its shelf life	[70]
Corn starch, GM (*Delonix regia* seeds), glycerol, and vanillin	Coatings Coating materials were used to protect climacteric fruits (*D’Anjou* pears)	[71]
GM (*Caesalpinia pulcherrima* seed), *Cymbopogon citratus* essential oil, and polysorbate	Coatings Coating materials were applied in *coalho* cheese	[72]
GM (*Gleditsia triacanthos* seeds), glycerol, and corn oil	Coating Coating materials reduced weight loss in “Regional” cheese, extending its shelf life	[73]
GM fenugreek (*Trigonella foenum-graecum*) and GG (*Cyamopsis tetragonoloba*), olive oil, and glycerol	Coating Coating materials maintained the postharvest quality of guava (*Psidium guajava* L.)	[32]
Carnauba wax, shellac, oleic acid, xanthan gum or GG (*Cyamopsis tetragonoloba*) or LBG (*Ceratonia siliqua*), morpholine, thiabendazole, and imazalil	Coating Wax‐hydrocolloid coatings that improve the shelf life of mandarins (*Citrus reticulata blanco*) without impairing the biochemical physiology of the fruit	[36]
Chitosan/dialdehyde GG (*Cyamopsis tetragonoloba*) hydrogels containing pomegranate (*Punica granatum* L.) peel extract	Hydrogel was observed to have a good potential to be used as an antibacterial pad for absorbing liquid at the bottom of trays of fresh meat, chicken, and fish packages These materials were not applied in food systems	[26]

**Table 2:** Mechanical properties and contact angle of galactomannan (GM)‐based films.

**GM source**	**M/G ratio**	**Formulation**	**TS (MPa)**	**EB (%)**	**EM (MPa)**	**WCA (°)**	**Ref.**
*Gleditsia triacanthos*	2.5:1	‐GM ‐Gly 50%	7.79	33.7	1.6	55	[61]
		‐GM 30% ‐SPI 70% ‐Gly 50%	2.58	27.4	8.3	73	
		‐GM 50% ‐SPI 50% ‐Gly 50%	3.72	38.0	7.1	56	
TG (*Caesalpinia spinosa*)	3.03:1	‐GM ‐Gly 30%	20.71	21.6	N.i.	≈55	[38]
		‐GM 90% ‐PVA 10% ‐Gly 30%	26.56	4.10	N.i.	≈80	
		‐GM 80% ‐PVA 20% ‐Gly 30%	39.29	6.01	N.i.	≈80	
		‐GM 70% ‐PVA 30% ‐Gly 30%	44.74	6.62	N.i.	≈90	
*Adenanthera pavonina*	1.3:1	‐GM ‐Gly 33%	6.5	35	N.i.	65.2	[59]
GG	1.7:1	‐GM ‐Gly 33%	3.7	50	N.i.	82.9	
*Caesalpinia pulcherrima*	2.9:1	‐GM ‐Gly 33%	2.5	70	N.i.	96.0	
LBG	3.4:1	‐GM ‐Gly 33%	1.3	50	N.i.	76.4	
*Sophora japonica*	5.6:1	‐GM ‐Gly 33%	7.0	45	N.i.	65.2	
Galactomannans recovered from spent coffee grounds	N.i.	‐BPHA	0.3	13.2	4.8	N.i.	[72]
	N.i.	‐BPHAE	0.2	5.3	8.5	N.i.	
*Cassia grandis* seeds	2.45:1	‐GM ‐Gly 25%	4.7	18.1	0.3	68.7	[74]
		‐GM ‐Lf 0.1% ‐Gly 25%	10.1	3.8	4.6	123.0	
		‐GM ‐Lf 0.2% ‐Gly 25%	9.4	3.4	5.1	122.8	
		‐GM ‐Lowpept 0.1% ‐Gly 25%	8.1	3.6	3.8	83.6	
		‐GM ‐Lowpept 0.2% ‐Gly 25%	11.4	3.2	6.2	93.1	
		‐GM ‐Lowcol 0.1% ‐Gly 25%	10.58	2.36	6.77	67.34	
		‐GM ‐Lowcol 0.2% ‐Gly 25%	8.25	3.29	4.51	62.06	

Abbreviations: EB, elongation at break; EM, elastic modulus; M/G, mannose/galactose; TS, tensile strength; WCA, water contact angle.

**Table 3:** Water contact angle, moisture content, and total soluble matter of galactomannan (GM) films.

**GM source**	**M/G ratio**	**Formulation**	**WCA (°)**	**MC (%)**	**TSM (%)**	**Reference**
*Gleditsia triacanthos*	2.5:1	‐GM ‐Gly 50%	55	34.41	89.7	[54]
		‐GM 30% ‐SPI 70% ‐Gly 50%	73	30.67	39.4	
		‐GM 50% ‐SPI 50% ‐Gly 50%	56	33.77	58.7	
*Delonix regia seeds*	3.5:1	‐GM ‐Gly 40% ‐Vanillin 10%	N.i.	23.5	66.9	[50]
		‐GM ‐Gly 40%	N.i.	24.9	63.0	
		‐GM ‐Vanillin 10%	N.i.	14.5	65.8	
		‐GM	N.i.	13.9	79.0	
		‐GM 50% ‐SBCS 50% ‐Gly 40% ‐Vanillin 10%	N.i.	23.9	66.0	
		‐GM 50% ‐SBCS 50% ‐Gly 40%	N.i.	22.6	62.8	
		‐GM 50% ‐SBCS 50% ‐Vanillin 10%	N.i.	13.7	58.2	
		‐GM 50% ‐SBCS 50%	N.i.	15.3	61.5	
*Adenanthera pavonina*	1.3:1	‐GM ‐Gly 33%	65.2	9.1	64.4	[39]
GG	1.7:1	‐GM ‐Gly 33%	82.9	7.8	81.1	
*Caesalpinia pulcherrima*	2.9:1	‐GM ‐Gly 33%	96.0	6.8	92.8	
LBG	3.4:1	‐GM ‐Gly 33%	76.4	9.0	26.8	
*Sophora japonica*	5.6:1	‐GM ‐Gly 33%	65.2	8.0	44.4	
*Cassia grandis* seeds	2.45:1	‐GM ‐Gly 25%	68.7	29.3	74.0	[58]
		‐GM ‐Lf 0.1% ‐Gly 25%	123.0	17.7	55.7	
		‐GM ‐Lf 0.2% ‐Gly 25%	122.8	15.7	51.7	
		‐GM ‐Lowpept 0.1% ‐Gly 25%	83.6	31.7	69.7	
		‐GM ‐Lowpept 0.2% ‐Gly 25%	93.1	26.3	63.7	
		‐GM ‐Lowcol 0.1% ‐Gly 25%	67.34	20.33	69.33	
		‐GM ‐Lowcol 0.2% ‐Gly 25%	62.06	18.00	56.00	

Abbreviations: MC, moisture content; M/G, mannose/galactose; TSM, total soluble matter; WCA, water contact angle.

**Table 4:** Gas and water vapor permeability of galactomannan (GM)‐based films.

**GM source**	**M/G ratio**	**Formulation**	**W** **V** **P** × 10^−10^ **(g·[s·m·Pa]** ^**−1**^ **)**	***P***_**O2**_ **(cm** ^**3**^ **·mm·[m** ^**2**^ **·atm·day]** ^**−1**^ **)**	**P**_**C** **O**2_ × 10^−15^ **(g·m·[Pa·s·m** ^**2**^]^**−1**^ **)**	**Ref.**
TG (*Caesalpinia spinosa*)	3.03:1	‐GM ‐Gly 30%	1.71	2.12	N.i.	[38]
		‐GM 90% ‐PVA 10% ‐Gly 30%	1.32	2.48	N.i.	
		‐GM 80% ‐PVA 20% ‐Gly 30%	0.91	2.24	N.i.	
		‐GM 70% ‐PVA 30% ‐Gly 30%	0.91	1.22	N.i.	
*Gleditsia triacanthos*	2.5:1	‐GM ‐Gly 100% ‐Corn oil 100%	39.93	1.39 × 10^−05^	34.88	[10]
		‐GM ‐Gly 133% ‐Corn oil 33%	32.40	8.12 × 10^−06^	15.35	
		‐GM ‐Corn oil 33%	26.90	2.10 × 10^−05^	12.84	
*Caesalpinia pulcherrima*	2.88:1	‐GM ‐Gly 200%	52.50	8.38 × 10^−06^	37.57	[9]
		‐GM ‐Gly 300%	62.50	8.55 × 10^−06^	28.81	
		‐GM ‐Gly 400%	77.00	9.50 × 10^−06^	4.10	
		‐GM ‐Gly 133%	51.20	8.99 × 10^−06^	14.95	
		‐GM ‐Gly 200%	53.30	4.32 × 10^−06^	47.85	
*Adenanthera pavonina*	1.35:1	‐GM ‐Gly 300%	69.80	4.58 × 10^−06^	17.40	
		‐GM ‐Gly 400%	81.00	7.43 × 10^−06^	10.94	
		‐GM ‐Gly 100%	50.20	3.20 × 10^−06^	43.13	
		‐GM ‐Gly 150%	64.70	3.72 × 10^−06^	29.29	
		‐GM ‐Gly 100%	48.90	2.68 × 10^−06^	61.19	
		‐GM ‐Gly 150%	61.80	3.54 × 10^−06^	47.08	
		‐GM ‐Gly 200%	68.10	4.23 × 10^−06^	30.26	
*Sesbania cannabina*	2.3:1	‐XGM	674.00	39.52	N.i.	[60]
		‐XGM ‐GO 0.5%	588.00	25.34	N.i.	
		‐XGM ‐GO 1%	506.00	23.30	N.i.	
		‐XGM ‐GO 2%	433.00	16.21	N.i.	
		‐XGM ‐GO 3%	405.00	15.20	N.i.	
		‐XGM ‐GO 5%	385.00	11.14	N.i.	
		‐XGM ‐GO 3% ‐PDMS coating	15.00	0.00	N.i.	
*Adenanthera pavonina*	1.3:1	‐GM ‐Gly 33%	67.8	2.41 × 10^−05^	28.8	[59]
GG	1.7:1	‐GM ‐Gly 33%	104.5	1.60 × 10^−05^	37.1	
*Caesalpinia pulcherrima*	2.9:1	‐GM ‐Gly 33%	106.9	1.42 × 10^−05^	42.9	
LBG	3.4:1	‐GM ‐Gly 33%	91.6	1.51 × 10^−05^	37.2	
*Sophora japonica*	5.6:1	‐GM ‐Gly 33%	80.6	2.54 × 10^−05^	28.7	

Abbreviations: M/G, mannose/galactose; *P*_CO2_, carbon dioxide permeability; *P*_O2_, oxygen permeability; WVP, water vapor permeability.

GM chemical modification is an alternative to produce films with better physicochemical properties. Films esterified through the anhydride esterification method before film formation (E‐GM) achieved notable properties. The E‐GM‐1.5 film (acetic anhydride to GM ratio of 1.5:1) displayed the highest esterification degree (0.05), hydrophobicity (107°), mechanical strength (92.0 MPa), and excellent oxygen barrier capabilities. Additionally, esterified films demonstrated lower toxicity to macrophage cells in vitro, supporting their suitability for food packaging applications sourced from natural materials [56].

Another approach to modification is through enzymatic treatment. The application of mannanase can enhance the tensile strength and elongation of GG films by reducing their degree of polymerization [57].

UV irradiation and chemical treatment (glutaraldehyde) of GM films enhance tensile strength, water vapor permeability, and flexibility for food packaging applications. Increasing doses of photoinitiator during UV irradiation progressively consume the free hydroxyl groups in GM, resulting in the formation of cross‐linked networks that decrease the hydrophilicity and water vapor permeability of the modified film. The incorporation of sodium benzoate improved the film’s strength, while the reaction with glutaraldehyde resulted in a more homogeneous surface, increased hydrophilicity, and enhanced flexibility, increasing elongation by nearly 15 times [58].

A different approach to improve the physicochemical film properties is incorporating nanomaterials. Nano–zinc oxide (nano‐ZnO) GM composite films exhibit superior tensile strength to high density polyethylene films and better oxygen barrier properties than polyvinyl chloride plastic film. They exhibit improved hydrophobicity, UV resistance, and antibacterial properties (against *E. coli* and *Bacillus subtilis*), offering potential for future alternatives in functional plastic food packaging [59].

Incorporating graphene oxide (GO) and poly(dimethylsiloxane) (PDMS) into GM films derived from *Sesbania cannabina* seeds results in hydrophobic surfaces with self‐cleaning properties. Inspired by the layered structure of nacre, an ultraflexible GM film cross‐linked with borate and GO was developed, capable of being molded into various shapes. This film achieved a tensile strength of 135.54 MPa, which is 2.4 times higher than that of pure GM film. Upon PDMS coating, the film became hydrophobic (WCA ≈ 120^°^) and self‐cleaning, also demonstrating enhanced oxygen and water vapor barrier properties [60].

The combined use of soy protein as a co‐component with the GM fraction from *Gleditsia triacanthos* seeds enhances the properties of edible films. This combination produced films with increased opacity, water resistance, and mechanical strength. GM reduced moisture absorption, hydrophilicity, and tensile strength, making the films suitable for food packaging applications [61]. Although some advances have been informed about the production and application of GM‐based films, further research is needed to investigate the stability of these materials against time and temperature, as well as their biodegradation and migration when exposed to food simulants.

### 4.2. Coatings

The use of renewable resources, particularly hydrocolloids derived from natural origins, has become a key focus in the packaging industry. Edible coatings are regarded as a promising technology to enhance food storage life and complement current packaging methods (Table 1). This approach contributes to maintaining microbial safety and protecting food from external influences [7].

Coatings formulated with GM and collagen have been shown to effectively reduce gas transfer rates in fruits, thereby slowing down respiration and delaying ripening processes. This innovative approach can significantly extend the shelf life of fresh produce while maintaining its quality, offering a natural and sustainable solution for postharvest preservation (Table 3) [62].

GM coatings have been tested on several tropical fruits, such as acerola, mango, pitanga, and seriguela. The ideal composition of the coatings varies according to the fruit, but generally includes GM in concentrations of 0.5%–1.5% and glycerol of 1.0%–2.0% [9]. GM‐based coatings have been shown to be effective in reducing weight loss, maintaining firmness, and preserving the color of the fruits during storage [63, 64].

Edible coatings based on GM are often combined with other components, such as collagen, glycerol, and carnauba wax, to improve their physical and chemical properties. Studies have shown that combining GM with collagen and glycerol can significantly reduce gas transfer rates in fruits such as mangoes and apples, extending their shelf life [9, 62].

Coating guavas (*Psidium guajava* L. cv. Paluma) with carnauba wax and GM (*Caesalpinia pulcherrima*) effectively preserved their postharvest quality by maintaining firmness, color, and improved antioxidant capacity while preventing the occurrence of chilling injuries during refrigeration [63].

Another study also evaluated the application of guava coatings, however, with FSG and GG. Coatings with 1.24% GG and 1.01% FSG significantly reduced weight loss, maintained firmness, and improved shelf life of guava. Changes in TSS, pH, and acidity during storage further highlighted the effectiveness of the coatings in preserving fruit quality [64].

LBG and GG were tested in citrus fruit wax‐hydrocolloid‐based coatings (mandarins of the cultivars Nova and Michal), along with xanthan gum for comparison. Both GMs effectively reduced weight loss during respiration, similar to standard wax coatings. LBG wax coatings improved juice flavor quality and altered the wax structure, improving fruit respiration. LBG outperformed GG and was comparable to xanthan gum in overall performance [65].

Edible coatings using GM from *Adenanthera pavonina* and *Caesalpinia pulcherrima*, collagen, and glycerol reduced O_2_ consumption and CO_2_ production by 28% and 11% in mangoes and by ~50% in apples, highlighting their potential to extend fruit shelf life [62].

A study evaluated the effects of GM (*Gleditsia triacanthos*) and chitosan coatings, together with storage temperature, on gas exchange, weight, and quality of “Regional” cheese. The GM coating showed the most significant reduction in O_2_ consumption and CO_2_ production, decreased moisture and weight loss, and minimized color changes during storage. The GM coating also reduced hardness and supported the incorporation of natural preservatives, demonstrating its potential to extend the shelf life of cheese [66].

Ricotta cheese coated with GM from *Gleditsia triacanthos* and nisin (50 IU/g) significantly reduced the growth of *L. monocytogenes* for 28 days at 4°C. The coating with added nisin improved film properties, including lower O_2_ permeability, higher CO_2_ permeability, tensile strength, and elongation [67].

GM coatings have good permeability properties to water vapor, oxygen, and carbon dioxide, as well as tensile strength and elongation at break. These properties are essential for the effectiveness of coatings in food preservation (Table 4) [7, 9].

In addition to fresh food applications, cross‐linked LBG (XGM) has been explored as coating materials for controlled drug release in the colon. High‐swelling XGM coatings showed faster disintegration but lower mechanical stability, with internal stress causing small ruptures. It is suitable for targeted drug release, with adjustable dissolution rates according to enzyme activity, although optimal performance has not been fully achieved [68].

## 5. Future Trends

GM can be used in the production of edible films/coatings for food applications, potentially replacing nonbiodegradable/nonedible plastics in the near future [7]. Particularly, most research studies have focused on the application of GM isolated from GG, LBG, TG, and FSG to manufacture packaging materials (Figure 1). A key future objective in GM research is the development of materials with enhanced mechanical and barrier properties to meet the demands of modern food packaging. Strategies such as UV irradiation and chemical treatments have proven effective in improving the tensile strength, water vapor permeability, and flexibility of GM‐based films. These modifications not only enhance their performance but also expand their potential applications, paving the way for sustainable, high‐quality packaging solutions derived from natural resources [58].

Ongoing research focuses on enhancing the properties of GM through modifications such as esterification (E‐GM). This process has been shown to produce films with significantly improved hydrophobicity, exceptional oxygen barrier performance, and increased tensile strength [56].

Although esterification and other chemical modifications have been shown to increase the mechanical strength of GM films, achieving consistent and reliable tensile strength remains a significant challenge. For example, esterified GM films often exhibit improved mechanical properties, but the degree of improvement depends heavily on factors such as the type of esterifying agent, reaction conditions, and extent of modification. This variability highlights the need for further optimization and standardization in modification processes to ensure reproducible results and broader applicability in food packaging and other fields [56, 58].

GM films typically offer excellent oxygen barrier properties, making them suitable for various packaging applications. However, their high water vapor permeability poses a limitation, reducing their effectiveness in environments where moisture resistance is critical. Strategies such as UV irradiation and the incorporation of hydrophobic agents have shown potential in mitigating this issue by lowering water vapor permeability. Nevertheless, these methods require further refinement to achieve optimal performance, ensuring the films maintain a balance between moisture resistance, mechanical integrity, and overall functionality [58, 60].

GM films have the potential to be functionalized with active compounds such as antimicrobials and antioxidants, enhancing their ability to preserve food quality and safety. However, achieving a uniform distribution and stable incorporation of these compounds within the film matrix remains a significant challenge. Variations in dispersion can lead to inconsistent performance, while the stability and efficacy of the active compounds over the packaging’s shelf life require further investigation to ensure reliable and long‐lasting protective effects. Advances in formulation and processing techniques will be essential to address these limitations and unlock the full potential of GM‐based active packaging [7, 59].

The preparation of GM solutions can be challenging due to its complex structure and high molecular weight [25, 29]. A study was conducted to explore a new methodology to produce GM films through the casting technique, since the conventional approach typically involves dispersion in water using heaters and mechanical agitators. The proposed method used an industrial blender to disperse GG at room temperature. The results demonstrated that this alternative not only is viable in reducing production time and costs but also produces films with improved visual quality [69]. Further studies should be developed to evaluate the dispersivity of other components of this matrix.

Another promising approach to enhancing the properties of GM‐based films is the formulation of biocomposites. For instance, incorporating nano‐ZnO or Laponite into GM matrices results in food packaging films with significantly improved hydrophobicity, UV resistance, and antibacterial properties. These advancements not only enhance the functionality and durability of the films but also position them as a viable alternative to conventional plastic packaging, aligning with the growing demand for sustainable and multifunctional packaging solutions in the food industry [59, 70].

Concerning the use of GM for packaging applications, it is necessary to understand the biodegradability of GM‐based films and their mixtures with other ingredients to manufacture composite materials, as well as the life cycle assessment of GM‐based materials. In the same way, more information about the stability against time and temperature, as well as their potential migration when exposed to food simulants or directly on foods, must be investigated. The scale‐up of biopolymer food packaging is another often neglected topic, not just for GM [71]. In this way, it is important to investigate the production of GM‐based films by extrusion and thermoforming molding, as well as the impact of these production methods on the physicochemical properties of the resulting GM‐based materials. Recently, Ramos et al. [72] compared two methods for producing GM‐based films. The authors produced GM‐based films using a conventional protocol where GM was heated in a water bath at 80°C for 135 min. An unconventional method where GM was dispersed in water using an industrial blender at 20°C for 15 min was investigated. In both methodologies, GM‐based films were produced by the casting method, and the authors concluded that the industrial blender can be used to disperse GM in water, avoiding the use of a water bath. The same authors concluded that this new methodology can reduce the cost of film production, aiming at industrial applications.

Future trends emphasize the exploration of alternative sources GM to diversify their applications and enhance sustainability. For instance, GM extracted from spent coffee grounds presents a promising renewable resource for the production of biopolymeric films. These films exhibit tunable mechanical properties and water vapor permeability, making them suitable for biodegradable packaging solutions. This approach not only reduces waste by valorizing agroindustrial byproducts but also supports the development of eco‐friendly materials aligned with the principles of a circular economy [73].

## 6. Conclusions

GM films exhibit excellent potential for food packaging, offering strength, flexibility, and effective moisture and gas barriers. Incorporating nanomaterials or blending with natural polymers enhances their mechanical properties, moisture resistance, and UV protection. Modifications such as UV irradiation and chemical treatments further reduce hydrophilicity and water vapor permeability, improving food preservation. Functionalization with antimicrobial agents or antioxidants enables the development of edible films that inhibit bacterial growth and extend shelf life. As biodegradable alternatives to conventional plastics, GM films support the industry’s transition to sustainable materials. However, challenges remain in optimizing their mechanical and barrier properties and understanding their biodegradation under different conditions. Continued research is crucial to refining their performance and expanding their commercial applications in food packaging.

## Conflicts of Interest

The authors declare no conflicts of interest.

## Funding

This study was supported by the Conselho Nacional de Desenvolvimento Científico e Tecnológico (10.13039/501100003593, 302434/2022‐4) and the Coordenação de Aperfeiçoamento de Pessoal de Nível Superior (10.13039/501100002322).

## Data Availability

Results will be available on request.
